# Preferential Inhibition of Frontal-to-Parietal Feedback Connectivity Is a Neurophysiologic Correlate of General Anesthesia in Surgical Patients

**DOI:** 10.1371/journal.pone.0025155

**Published:** 2011-10-05

**Authors:** Seung-Woo Ku, UnCheol Lee, Gyu-Jeong Noh, In-Gu Jun, George A. Mashour

**Affiliations:** 1 Department of Anesthesiology and Pain Medicine, Asan Medical Center, University of Ulsan College of Medicine, Seoul, Korea; 2 Department of Anesthesiology, University of Michigan Medical School, Ann Arbor, Michigan, United States of America; University of British Columbia, Canada

## Abstract

**Background:**

The precise mechanism and optimal measure of anesthetic-induced unconsciousness has yet to be elucidated. Preferential inhibition of feedback connectivity from frontal to parietal brain networks is one potential neurophysiologic correlate, but has only been demonstrated in animals or under limited conditions in healthy volunteers.

**Methods and Findings:**

We recruited eighteen patients presenting for surgery under general anesthesia; electroencephalography of the frontal and parietal regions was acquired during (i) baseline consciousness, (ii) anesthetic induction with propofol or sevoflurane, (iii) general anesthesia, (iv) recovery of consciousness, and (v) post-recovery states. We used two measures of effective connectivity, evolutional map approach and symbolic transfer entropy, to analyze causal interactions of the frontal and parietal regions. The dominant feedback connectivity of the baseline conscious state was inhibited after anesthetic induction and during general anesthesia, resulting in reduced asymmetry of feedback and feedforward connections in the frontoparietal network. Dominant feedback connectivity returned when patients recovered from anesthesia. Both analytic techniques and both classes of anesthetics demonstrated similar results in this heterogeneous population of surgical patients.

**Conclusions:**

The disruption of dominant feedback connectivity in the frontoparietal network is a common neurophysiologic correlate of general anesthesia across two anesthetic classes and two analytic measures. This study represents a key translational step from the underlying cognitive neuroscience of consciousness to more sophisticated monitoring of anesthetic effects in human surgical patients.

## Introduction

Recent studies using neuroimaging, high-density electroencephalography (EEG) and transcranial magnetic stimulation have contributed significantly to our understanding of how general anesthetics might suppress consciousness [1]–[7]. However, such techniques are impractical for the routine intraoperative assessment of anesthetic depth in the approximately 40 million patients receiving general anesthetics each year in North America alone [8]. Conversely, currently available “awareness monitors” are practical for routine use, but employ empirically-derived algorithms that are not grounded in the cognitive neuroscience of consciousness or general anesthesia [9]. These algorithms are often proprietary, which precludes the open scientific investigation that could improve the detection of intraoperative awareness or advance the mechanistic understanding of general anesthesia. Thus, identifying a neural correlate or cause of consciousness that can be measured routinely in surgical patients would be an important translational advance.

Visual processing follows a posterior-to-anterior path from primary visual cortex to the temporal lobe (ventral stream) and frontal lobe (dorsal stream). However, evoked activity in the primary visual cortex and subsequent feedforward processing is not sufficient to generate conscious experience—a “feedback” pathway is also thought to be required [10]–[14]. Feedback processing has been discussed as a neural correlate of consciousness beyond the visual system [15]. Consistent with this possibility, preliminary evidence suggests that anesthetic-induced unconsciousness is associated with a selective inhibition of anterior-to-posterior feedback activity.

By measuring the transfer entropy of visual-evoked potentials in rats, Imas *et al* found that wakefulness was characterized by a balance of feedforward and feedback connectivity [16]. After general anesthesia was induced with the inhaled anesthetic isoflurane, feedback activity was selectively suppressed in association with a surrogate of anesthetic-induced unconsciousness. These data were supported by a later study of anterior-posterior phase synchronization [17]. We studied the directionality of frontoparietal connectivity during consciousness, propofol anesthesia, and recovery in human volunteers [18], [19]. Human subjects differed from rodents in that feedback connectivity was dominant in the conscious state. After induction with propofol, both feedforward and feedback connectivity precipitously decreased but feedforward connectivity recovered to baseline during general anesthesia, while feedback was suppressed until the return of consciousness. This study was limited in that it was conducted only in young healthy males receiving a bolus dose of a single intravenous anesthetic.

In the current study we tested the hypothesis that preferential inhibition of frontoparietal feedback connectivity is a common feature of general anesthesia in surgical patients. Using measures of effective connectivity, we demonstrate that frontoparietal feedback is reduced in patients receiving both inhaled and intravenous anesthetics and returns upon recovery. This study supports frontoparietal feedback connectivity as a neurophysiologic correlate of consciousness in humans and the preferential inhibition of such connectivity as a correlate of general anesthesia that could potentially be measured in the intraoperative setting.

## Results

### Two different analytic methods demonstrate that preferential inhibition of feedback connectivity is a neurophysiologic correlate of anesthetic-induced unconsciousness

**Figure 1:**
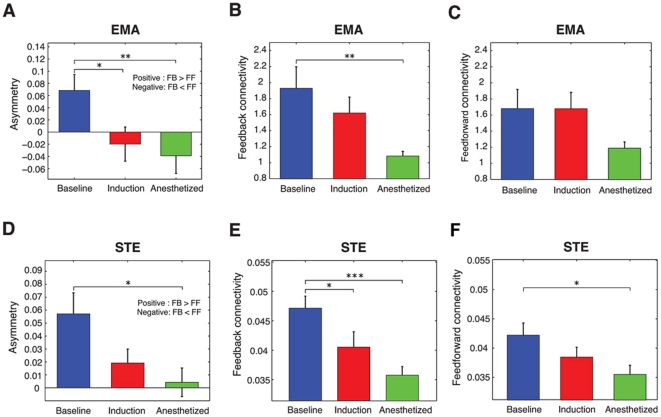
Feedback and feedforward connectivity in the frontoparietal network calculated by the evolutional map approach (EMA) and symbolic transfer entropy (STE). (A) The asymmetry between feedback and feedforward connectivity in the three states (baseline, induction and anesthetized) using the EMA method. (B–C) Absolute values of feedback (B) and feedforward (C) connectivity across the three states. The feedback dominance in the baseline was reduced due to inhibition of feedback phase modulation after induction. (D) The asymmetry between the feedback and feedforward connectivity in the three states (baseline, induction and anesthetized) using the STE method. (E–F) Absolute values for feedback (E) and feedforward (F) STE across the three states. The feedback dominance in the baseline state was reduced by inhibition of feedback STE after induction. However, feedforward STE values were also reduced in the anesthetized state. The errorbar denotes the standard error (*: p<0.05, **: p<0.01, ***: p<0.001, n = 18 patients).

Eighteen surgical patients receiving general anesthesia with propofol or sevoflurane were recruited for the study. Patient characteristics and case information are shown in Table 1; states of consciousness analyzed in this study are shown in Table 2. We used the evolutional map approach (EMA) and symbolic transfer entropy (STE) method, which are based on the different theoretical backgrounds of phase dynamics and information theory, to quantify the causal relationships between EEG of frontal and parietal regions. Figure 1 shows the average feedback and feedforward connectivity and its asymmetry measured by the EMA and STE methods. Eight pairs of EEG channels between the two regions (Fp1, Fp2, F3, F4 and P3, P4) were used for the calculation of bidirectional frontal-parietal connectivity. During baseline consciousness, there was asymmetry of feedback and feedforward connectivity (Figure 1A and 1D). By definition, the positive value of asymmetry in both measures indicates that feedback connectivity exceeds feedforward connectivity. After induction of anesthesia, the asymmetry was significantly reduced as assessed by EMA (Figure 1A): p = 0.0052, F = 6.166, df = 2 (states) and 17 (individuals), n = 18; repeated measures one-way analysis of variance [ANOVA] with Tukey's multiple comparison test: p<0.05 for baseline & induction, p<0.01 for baseline & anesthetized). Figure 1B and 1C demonstrate the individual means of feedback and feedforward connectivity, respectively, measured by the EMA method over three states. The feedback connectivity during baseline consciousness significantly decreased in the anesthetized state (p = 0.0083, F = 5.532, df = 2 (states), 17 (individuals), n = 18; repeated measures one-way ANOVA with Tukey's multiple comparison test: p<0.01 for baseline & anesthetized), while no significant difference in feedforward connectivity was found.

**Table 1:** Patient Characteristics.

	Propofol Group (n = 9)	Sevoflurane Group (n = 9)	All Patients (n = 18)
**Age (years)** *	50.3±9.0	42.7±8.2	46.5±9.2
**Sex (m/f)**	4/5	4/5	8/10
**Height (cm)** *	161.9±5.2	164.8±64.8	163.3±8.7
**Weight (kg)** *	60.8±6.3	67.2±7.2 t	64.0±9.5
**BMI (kg/m^2^)** *	23.2±2.1	24.9±3.7	24.1±3.1
**Type of surgery (number)**	Gastrectomy (6) Mastectomy (2) Gastrojejunostomy (1)	Gastrectomy (7) Mastectomy (1) Liver segmentectomy (1)	Gastrectomy (13) Mastectomy (3) Gastrojejunostomy (1) Liver segmentectomy (1)

*mean ± standard deviation. m = male; f = female; BMI = body mass index.

**Table 2:** Monitoring Epochs used for Analysis.

*States*	Start of Epoch	End of Epoch
**Baseline**	Before anesthetic induction, in the operating room, fully conscious	Five minutes after start of recording, fully conscious
**Induction**	Start of anesthetic induction	Loss of consciousness
**Anesthetized**	Loss of consciousness	Five minutes after loss of consciousness
**Recovery**	End of anesthetic maintenance	Recovery of consciousness
**Post-recovery**	Admission to recovery room	Five minutes after admission

Figure 1D presents feedback and feedforward connectivity measured by the STE method. The same procedure was applied to the EEG data as was performed with the EMA method. The mean of asymmetry and the individual means of feedback and feedforward information flow are presented in Figure 1D–1F. Like EMA in the baseline, the feedback information flow was dominant with a significantly larger positive value in asymmetry (Figure 1D). This large asymmetric information flow was reduced in the anesthetized state (p = 0.0295, F = 3.914, df = 2 (states), 17 (individuals), n = 18; repeated measures one-way ANOVA with Tukey's multiple comparison test: p<0.05 for baseline & anesthetized), resulting in balanced information flows across two directions. The reduced asymmetry was caused by a reduction of feedback connectivity, even though there was also a significant reduction in feedforward flow (Figure 1E and 1F); p = 0.0001, F = 11.72, df = 2 (states) and 17 (individuals), n = 18; repeated measures one-way ANOVA with Tukey's multiple comparison test: p<0.05 for baseline & induction, p<0.001 for baseline & anesthetized). In contrast to the EMA method, the STE method detected significant suppression of feedback connectivity during anesthetic induction (p = 0.0156, F = 4.711, df = 2 (states) and 17 (individuals), n = 18; repeated measures one-way ANOVA with Tukey's multiple comparison test: p<0.05 for baseline & induction).

### Two different classes of anesthetic cause preferential inhibition of feedback connectivity

**Figure 2:**
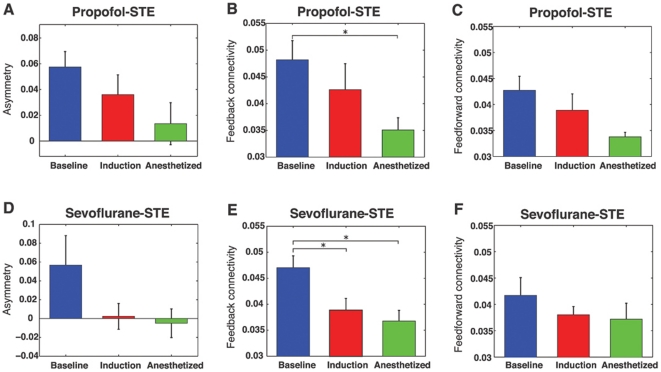
Analysis of asymmetry, feedback and feedforward symbolic transfer entropy (STE) for propofol and sevoflurane groups. (A) The asymmetry between (B) feedback and (C) feedforward STE for the propofol group (n = 9 patients). (D) The asymmetry between (E) feedback and (F) feedforward STE for the sevoflurane group (n = 9 patients). The error bar denotes the standard error (*: p<0.05). The results of the individual anesthetics are consistent with the combined data.

The effects of propofol and sevoflurane on feedback inhibition were analyzed individually using both the EMA and STE methods. The EMA method did not show any significant results because of large individual variances over the three states, although trends were consistent with the STE method. The feedback and feedforward information flows measured by STE for the individual anesthetics demonstrated similar results to those of the combined data (Figure 2A–2F). The dominant feedback information flow during consciousness and the symmetrical flow during general anesthesia due to reduction of feedback connectivity were found for both anesthetics (for feedback connectivity during propofol: p = 0.0167, F = 5.345, df = 2 (states) and 8 (individuals), n = 9; repeated measures one-way ANOVA with Tukey's multiple comparison test: p<0.05 for baseline & anesthetized; for feedback connectivity during sevoflurane: p = 0.004, F = 7.946, df = 2(states) and 8 (indivisuals), n = 9; repeated measures one-way ANOVA with Tukey's multiple comparison test: p<0.05 for baseline & induction, p<0.001 for baseline & anesthetized). One observed difference was that sevoflurane produced a balanced information flow during anesthetic induction. This preceded the effect of propofol, which resulted in balanced information flow during the anesthetized state. These differences may be due to the fact that equisedative concentrations were not being delivered during induction.

### Feedback connectivity increases during recovery from general anesthesia

**Figure 3:**
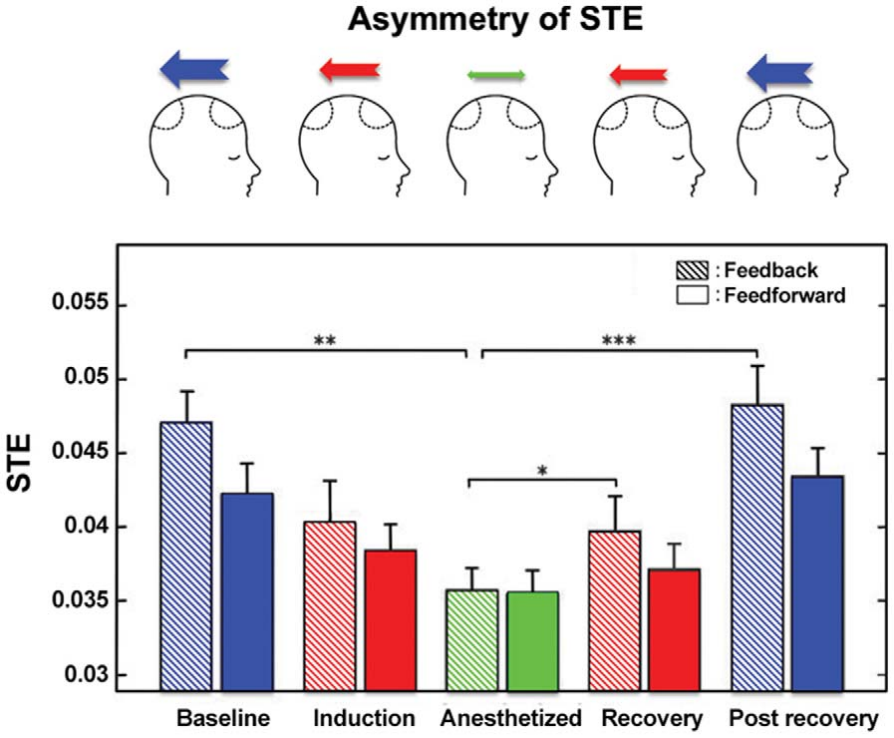
Post-anesthetic recovery of feedback symbolic transfer entropy (STE). The schematic diagrams in the top row represent the changing asymmetry between feedback and feedforward STE over the five states. A significant change in feedforward STE occurred only between anesthetized and post-recovery states (which is not presented in this figure). The feedback and feedforward STE are denoted with striped and solid colors, respectively, for each state. Error bar denotes the standard error (*: p<0.05, **: p<0.01,***: p<0.001, n = 18 patients).

Figure 3 demonstrates the return of dominant feedback connectivity measured by STE in the recovery and post-recovery state. The symmetric information flow during general anesthesia was disrupted during the recovery period (when drug administration was terminated), but was not significant yet. The asymmetric feedback and feedforward information flows returned to the baseline level in the post-recovery state. (For feedback: p = 0.0002, F = 6.294, df = 4 (states) and 17 (individuals), n = 18; repeated measures one-way ANOVA with Tukey's multiple comparison test: p<0.01 for baseline & anesthetized, p<0.0001 for anesthetized & post-recovery, p<0.05 for recovery & post-recovery; For feedforward: p = 0.0059, F = 3.976, df = 4 (states) and 17 (individuals), n = 18; repeated measures one-way ANOVA with Tukey's multiple comparison test: p<0.05 for anesthetized & post-recovery). By contrast, the EMA did not show significant recovery of connectivity in the post-recovery state because of large variance. In this study the recovery and post-recovery states were excluded from the main analysis because the effects of different surgical procedures and analgesic interventions on feedback and feedforward connectivity could not be estimated.

### Preferential inhibition of feedback connectivity is not attributable to spectral changes

The potential spurious feedback and feedforward connectivity derived from the difference of power spectra between frontal (Fp1, Fp2 and F3 and F4) and parietal (P3 and P4) regions was estimated by using the surrogate data method. Surrogate data maintains the original power spectra of EEG, but phase information is randomized. Thus, EMA and STE measures of frontoparietal feedback connectivity using surrogate data should theoretically have values of zero; non-zero values provide an estimate of spurious causality derived from a difference of power spectra between two brain regions.

**Figure 4:**
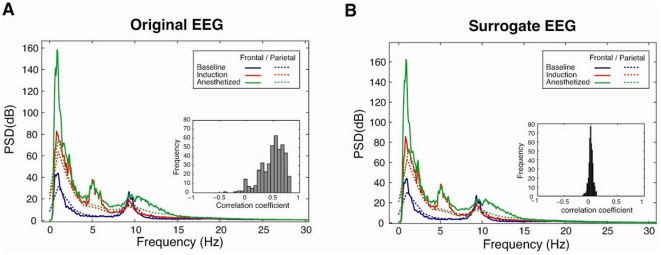
Power spectra and correlations of the frontal and parietal regions for the original and surrogate EEG. The original (A) and surrogate EEG data (B) have the same power spectral densities for the frontal (solid lines) and parietal (dotted lines) regions for three states (red: baseline, blue: induction, green: anesthetized). The distribution of linear correlation coefficients (the zeroth lag of the normalized covariance function) between frontal and parietal EEG channels has a positive mean value (Inset in (A)), whereas the distribution for surrogate data has a zero mean value (Inset in (B)). n = 18 patients.

Figure 4A shows the average power spectral densities of the frontal (solid lines) and parietal (dotted lines) EEG data, whereas Figure 4B shows the average power spectral densities of the surrogate data of the frontal and parietal EEG. The insets of Figure 4A and 4B demonstrate the histograms of linear correlation coefficients (the zero lag of the normalized covariance function) between frontal and parietal regions over 18 patients for the original EEG and surrogate data sets. The surrogate data set has the same power spectra with that of the frontal and parietal EEGs for the baseline, induction and anesthetized states. The distribution of correlation coefficients of the original EEG data between frontal and parietal regions has a large positive mean (inset of Figure 4A). As would be predicted, the distribution of correlation coefficients for the surrogate data has a mean of zero (inset of Figure 4B). Anesthetic induction generated increased power of lower frequency bands, particularly in the frontal region, which is a typical spectral change in the anesthetized state.

**Figure 5:**
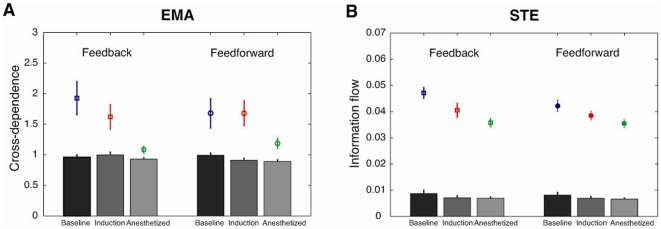
Estimation of bias caused by power spectral differences between frontal and parietal regions. The biases caused by the power spectral difference between frontal and parietal regions were denoted with mean and standard error over 18 patients in for EMA (A) and STE (B). Connectivity measures based on the original EEG data (feedback-squares, feedforward-circles) show that the biases do not account for changes across states. n = 18 patients.

Figure 5A and 5B show the feedback and feedforward connections measured by EMA and STE using the surrogate data. The surrogate data of frontal and parietal EEG have non-zero EMA and STE values, which reflect estimates of spurious feedback and feedforward measures due to spectral changes. However, the bias based on the power spectra does not fully account for the EMA and STE values measured in the original data and furthermore does not change across states.

## Discussion

This is the first study to demonstrate that preferential inhibition of frontoparietal feedback connectivity is a clinically-relevant neurophysiologic correlate of general anesthesia in surgical patients. These results are consistent with our prior findings in humans [18], but the current data are significantly more generalizable to the perioperative setting since feedback connectivity inhibition was shown across two different classes of anesthetics, two analytic techniques, and a heterogeneous mix of patients. Furthermore, past studies were performed with a single bolus injection as opposed to continuous target-controlled infusion or graded inhalational induction. Frontoparietal feedback connectivity was preferentially reduced after the administration of general anesthetics, thereby reducing the asymmetry of information flow; importantly, it was found to return at full recovery from anesthesia. Thus, analysis of frontoparietal feedback connectivity in relatively few EEG channels may be able to distinguish different phases of surgical anesthesia, especially using the STE method. These data are impactful because they suggest that cognitive neuroscientific principles of consciousness can potentially be measured with routine technology in the perioperative setting. Importantly, these findings are consistent with recent data demonstrating that vegetative states are also associated with loss of top-down, feedback connectivity across frontal and temporal lobes [20].

It has been suggested that feedforward projections represent incoming sensory data, whereas feedback projections play a modulating role in the selection and contextual interpretation of information [21]–[23]. The preferential inhibition of feedback connectivity is consistent with other findings suggesting that primary sensory processing and local sensory networks are preserved during anesthesia, while higher-order information synthesis is inhibited [2], [3], [24], [25]. The finding that feedforward activity largely persists during induction and in the anesthetized state by the EMA method and during induction by the STE method may represent such primary pathways of sensory processing. However, the STE method detected a significant reduction in feedforward connectivity in the anesthetized state, in addition to the reduction of feedback connectivity (Figure 2B and 2C). The decrease of both directional connections was reported in animal studies at the plane of surgical anesthesia and also appeared transiently after injection of a propofol bolus in human subjects [16], [18]. This may be due to significant disruption of phase synchronization between frontal and parietal regions [17] as well as disruption of optimal functional networks in the parietal region [26]. It is also possible that the addition of opiates after induction of anesthesia resulted in a reduction of incoming sensory information.

Most methods for detecting causal relationship between signals have the potential to generate spurious causality. Differences of dynamic structures, noise color, and noise intensity for two systems under study are possible sources of spurious causality [27], [28]. Therefore, we employed two different approaches based on phase dynamics (EMA) and information theory (STE), each of which has specific strengths and weaknesses. The fact that the preferential inhibition of frontoparietal feedback during general anesthesia was consistent across two methods suggests that it is a robust finding. Furthermore, we controlled for spurious feedback and feedforward connections in EMA and STE (Figure 5) attributable to power spectral differences by using the surrogate data method. As a result, we confirmed that differences in feedback connectivity during general anesthesia were not solely attributable to changes in spectral contents. Based on this analysis, STE appears to be less susceptible than EMA to spurious causality, showing relatively lower biases (Figure 4D).

There are numerous limitations to our study. First, eight EEG channels have low spatial resolution and did not cover all frontal and parietal regions. However, the goal of this study was to use a relatively low number of channels to enhance the translational impact of the study. Second, we did not consider the posterior region, which could yield important information about the effects of general anesthetics [29]. However, the goal was to focus on frontoparietal interactions during anesthesia, which are physically more accessible for measurement in surgical patients. Third, there was a limited number of patients for each anesthetic in this study. Fourth, current algorithms for detection of a causal relationship between signals cannot distinguish a causal effect from a common source input. For instance, if a common source drives the frontal and parietal regions with a time delay, it could appear that there was a causal relationship. Similarly, volume conduction could result in spurious causality. Surrogate data analysis in the current study demonstrated a zero mean of cross-correlation; although analysis of frontoparietal causality in the surrogate dataset resulted in non-zero values for both analytic techniques (Figure 5), such spurious causality did not account for our primary findings across states of consciousness. Fifth, we assessed only nonlinear relationships between frontal and parietal regions. For example, Granger causality was not used in this study because it considers linear properties and is sensitive to parameter sets and preprocessing of EEG [30]. Sixth, the two anesthetics were not delivered at equipotent concentrations; the concentrations of sevoflurane and propofol were not at true steady-state. Steady-state concentrations require long intervals between transitions, which is not the case during routine clinical induction of anesthesia. Therefore, we focused on total induction duration of the two drugs. Seventh, analysis of causal relationships between frontal and parietal regions was calculated after the surgery. Further technological development and clinical study are required to see if these methods are effective as real-time monitors of frontoparietal connectivity. Finally, our study does not address the underlying neural mechanisms that cause the observed preferential inhibition of feedback connectivity during general anesthesia. Ongoing studies are being conducted to address this important question.

In conclusion, these data suggest that preferential inhibition of frontoparietal feedback connectivity is a neurophysiologic correlate of general anesthesia in a routine clinical setting. This translational study establishes a foundation for more sophisticated intraoperative monitoring as well as further investigation of anesthetic mechanisms in corticocortical networks.

## Materials and Methods

### Subjects

The study was approved by the Institutional Review Board of Asan Medical Center (Seoul, South Korea) and written informed consent was obtained in all cases; EEG data were analyzed at the University of Michigan Medical School (Ann Arbor, MI). Patients scheduled for elective abdominal or breast surgery (n = 18, male/female = 8/10, American Society Anesthesiologists Physical Status I or II, age 29–66 years) were enrolled in this study (See Table 1 for details). Exclusion criteria included a previous head injury with loss of consciousness, a previous brain surgery, a history of drug or alcohol dependence, known neurological or psychiatric disorders, or current use of psychotropic medications.

### Anesthetic procedures

Patients received no sedatives or other medications before induction of anesthesia. One of two anesthetic regimens was randomly selected and administered to eighteen patients: (i) Propofol (Diprivan®, AstraZeneca, London, UK), initially target-controlled infusion of propofol 2.0 µg/ml was started and increased at a rate 1.0 µg/ml per 20 s until loss of consciousness (LOC) for nine patients; or (ii) Sevoflurane (Sevorane®, Abbott, Illinois, USA), 2 vol% was started and increased at a rate 2 vol% per 20 s until LOC for the other nine patients. Time to LOC was determined by checking every 5 s for the loss of response to verbal command (“open your eyes”). It must be noted that interpretations of unconsciousness are necessarily subjective, as there is no established standard to differentiate LOC from merely loss of responsiveness. If patients were not able to ventilate spontaneously due to the effects of propofol or sevoflurane, their lungs were manually ventilated with 100% oxygen via facemask, to maintain an end-tidal carbon dioxide tension of 35–45 mmHg.

After induction of general anesthesia, patients received an effect-site target propofol concentration of 3 µg/ml in combination with target remifentanil concentration of 5 ng/ml, or 2–3 vol% sevoflurane. Rocuronium (0.6 mg/kg) was administered to facilitate orotracheal intubation. After surgery, the time to recovery of consciousness (ROC) was monitored during emergence. The point of ROC was determined by the recovery of response to a verbal command (“open your eyes”) every 5 s. After regaining consciousness and spontaneous respiration, subjects were transferred to the Post-Anesthesia Care Unit breathing room air. EEG data were acquired throughout.

### Data acquisition

EEG was recorded at eight monopolar channels in the frontoparietal region (Fp1, Fp2, F3, F4, T3, T4, P3 and P4 referenced by A2, which followed the international 10–20 system for electrode placement) by a WEEG-32 (LXE3232-RF, Laxtha Inc., Daejeon, Korea) with a sampling frequency of 256 Hz. Electromyogram (EMG) was concurrently recorded at four bipolar channels (bilateral frontalis and temporalis muscle) by a QEMG-4 (Laxtha Inc., Daejeon, Korea) with a sampling frequency of 1024 Hz. The attached position of the four muscle electrode pairs followed Goncharova et. al. [31]. Patients were also monitored with electrocardiography, pulse oximetry, end-tidal carbon dioxide concentration and non-invasive blood pressure measurement.

The EEG and EMG recordings were divided into five monitoring epochs (Table 2): (i) baseline, 5 min before anesthetic induction; (ii) induction, from start of anesthetic induction to LOC; (iii) anesthetized state, 5 min after LOC; (iv) recovery, from the end of anesthesia to ROC; (v) post-recovery, 5 min after recovery in the Post-Anesthesia Care Unit. Recovery of patients in the Post-Anesthesia Care Unit was defined as an Observer's Assessment of Alertness/Sedation Scale value greater than 5.

To investigate the causal relationship between activity in frontal and parietal regions, one-minute-long artifact-free EEG epochs were selected by visual inspection among five-minute-long EEG epochs during the five states. We excluded EEG epochs coinciding with increase of EMG amplitude and containing non-stationary wave changes in one-minute EEG epochs. Fourier-based band-pass filtering (0.5–55 Hz) was applied to EEG data before the calculation of directionality.

### Quantitative analysis of frontoparietal feedforward and feedback connectivity

Feedforward and feedback connectivity in the frontal and parietal regions were quantified based on digitized EEG data. The basic concept of identifying causality between two signals was stated by Wiener in 1956 [32]: “For two simultaneously measured signals, if we can predict the first signal better by using the past information from the second one than by using the information without it, then we call the second signal causal to the first one.” The causal relationship between two signals of the EEG reflects a directed functional connection in the brain. For the purposes of this study, if the frontal activity was the cause of parietal activity, it was deemed a “feedback” connection; conversely, if the parietal activity was the cause of frontal activity, it was deemed a “feedforward” connection. For our systematic assessment of the directional flow of information in the frontoparietal system during consciousness and anesthesia, two methods based on different theoretical backgrounds were employed: (i) EMA, which is based on the phase dynamics of two signals [33], and (ii) STE, which is based on information theory [34].

### Evolutional Map Approach (EMA)

If we assume that two EEG signals 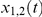 influence each other through weak coupling, then the weak coupling would be primarily manifested as an effect on the phases of EEG, rather than the amplitudes. EMA measures the cross-dependence of coupled nonlinear oscillators based on their phase dynamics [33]. The phases 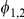 of signals 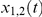 were obtained by Hilbert transformation, and the phase increments 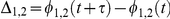 were calculated during time increment 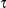. The influence of 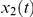 on 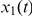 is estimated by the dependency of 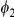 on 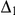. In practice, the phase increment was expressed as a function of phases 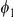 and 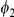 by finite Fourier series: 
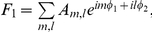
 
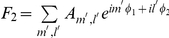
, where 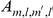 were the coefficients and 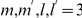 were set as optimal for our EEG.

The cross dependence between 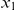 and 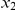 are calculated as followed:
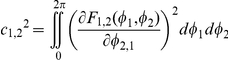
Here, 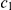 is the influence of 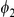 to 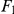 and 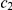 is vice-versa. 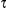 was set as 1 s, considering that the time required for conscious processing is thought to exceed 270 ms [35]. In order to avoid edge effects, the Hanning window (cosine half-wave) was applied to the beginning and the end of one-minute-long EEG data (1.5 s on each end). After applying the Hilbert transform, the phase values of 1.5 s were discarded on each side of the data. The reliability of the cross-dependence 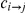 and 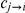 was tested with models and application to empirical data [33], [36]–[39].

The directed functional connectivity, 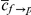, between two scalp areas was defined as average cross dependences from one to the other scalp areas in both directions, and the mean directionality index 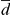 is a normalized form of the cross-dependences, which indicates the asymmetry of modulation:
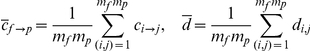
where 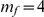 and 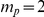 are the number of EEG channels on both scalp areas, respectively, and the index 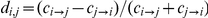 varies from 1 in the case of unidirectional coupling (*i*→*j*) to −1 in the opposite case (*j*→*i*) with intermediate values 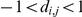 corresponding to bidirectional coupling.

### Symbolic Transfer Entropy (STE)

STE offers a nonlinear, model-free estimation of directional information flow based on information theory, quantifying the degree of dependence of *Y* on *X* or vice-versa among two signals *X* and *Y* [34], [40]. In contrast to EMA, STE considers the amplitudes as well as the phases of a signal. For a given two signals *X* and *Y*, if a present state 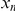 of signal *X* is a cause of future state 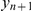 of signal *Y*, the two conditional probabilities, 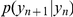 and 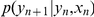, are different, whereas for the independent case they are equal because 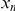 does not affect the future state 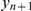. The Kullback-Leibler divergence quantifies the difference of two conditional probabilities. Therefore, the 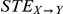 was defined as following,
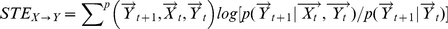
where 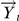 and 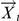 are the embedded vector points at time *t* with signal *Y* and *X*, respectively. For instance, 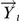 consists of the ranks of its components 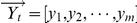, where 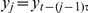 is replaced with the rank in ascending order, 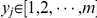 for *j* = 1, 2, 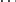, *m*. Here *m* is the embedding dimension and τ is the time delay. 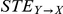 is defined in the same way, replacing *X* and *Y*. Therefore, if an EEG signal has influence on the other EEG signal, 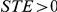, while if two signals are independent, 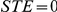.

The “feedback “and “feedforward” information flow, 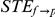 and 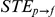, in the frontoparietal network were evaluated in the eighteen subjects. As proper embedding parameters for our data, *m* = 3 and 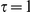 were chosen by searching the best parameter set. Thus, in this parameter set, 15,359 vector points were constructed for one-minute-long EEG data.

The average 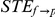 and 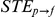 (Figure 1D–1F) was calculated over the eight pairs of EEG channels between frontal and parietal regions for each subject; 
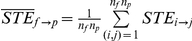
, where 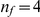 and 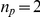. The asymmetry of information flow between two brain regions was defined as 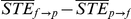 for each subject. Thus, positive values indicate the dominance of feedback connectivity, while negative values indicate the dominance of feedforward connectivity.

As compared with the original transfer entropy, STE is advantageous in that it avoids binning the measured values in the probability calculation. Furthermore, it is a more robust and computationally fast method to quantify the dominating direction of information flow between time series from structurally identical and non-identical coupled systems. The performance of this method has been validated in various applications [34], [40]–[42]. Furthermore, transfer entropy has been suggested to be a robust method for detecting true causal relationships in the setting of linear mixing of signals [42].

EMA and STE have different theoretical backgrounds: phase dynamics and information theory, respectively. As such, each method has its own set of advantages and disadvantages in the detection of causal relationships from EEG. By applying both methods to our EEG data, we could estimate the feedback and feedforward connectivity in the frontoparietal system during general in a more comprehensive way.

### Estimation of bias caused by differences in power spectra

One of the potential problems in estimating causal relationships is that spurious causality can result if two signals have significantly different spectral contents [17], [18], [23]. To estimate the amount of bias caused by power spectral differences for two EEG data sets, the surrogate data method was used. Surrogate data have precisely the same spectral contents as those of the original EEG data set, but their phases are randomly shuffled. Thus, we removed true connections by phase randomization between two EEG data sets; any non-zero value resulting from connectivity analysis would estimate bias caused by power spectral differences. To generate the surrogate data, the amplitude spectrum and amplitude distribution adjustment method was used [43]. Twenty surrogate data sets were generated for each minute of EEG data. The average feedforward and feedback connections using EMA and STE were estimated with 160 pairs of surrogate data for eight pairs of EEG channels between the frontal and parietal regions.

The average power spectral density was computed based on the Welch spectral estimator (MATLAB signal processing toolbox, “psd.m’ with options: ‘spectrum.welch’ with Hamming window and window size of 256). The average power spectral densities for frontal (Fp1, Fp2, F3 and F4) and parietal (P3 and P4) regions across three states in 18 patients are demonstrated in Figure 4A. The average power spectral densities of the corresponding surrogate data are demonstrated in Figure 4B.

### Statistical analysis

The feedback and feedforward information transfer in the frontal and parietal regions was analyzed by two different methods (EMA and STE). For each subject the average feedback and feedforward information flow was calculated with eight pairs of EEG channels, and the change of the bidirectional connections was evaluated over eighteen subjects. The statistical significance of the anesthetic effect on the feedback and feedforward connections was assessed by a repeated measures one-way ANOVA and Tukey's multi-comparison test across the three states (baseline consciousness, anesthetic induction and general anesthesia). A *p* value<0.05 was considered significant. The mean± standard error (SEM) and the results of the *post hoc* test are shown. The D'Agostino & Pearson omnibus normality test was applied before performing the ANOVA test. A formal statistical consultation was obtained at the Center for Statistical Consultation and Research at the University of Michigan (Ann Arbor, MI) and the GraphPad Prism Version 5.01 (GraphPad Software Inc. San Diego CA) was used.
